# The role of legislation in K-12 school discipline: The silence of action

**DOI:** 10.3389/fpsyg.2022.916925

**Published:** 2022-07-28

**Authors:** Mengmeng Bo, Gift Chinemerem Onwubuya

**Affiliations:** Department of Teacher Education, Faculty of Education, Shanghai Normal University, Shanghai, China

**Keywords:** social constructionism, corporal punishment, interaction, legislation, power relations, teachers’ right to discipline students

## Abstract

Researchers have consistently identified the disparity between teachers’ practical and legal knowledge regarding teachers’ right to discipline students. However, few studies have investigated teachers’ construction processes that form construction outcomes, which would help navigate the role of legislation in school discipline. This study contributes to a holistic picture of the neglected disciplinary rights that teachers construct in teaching practice and their underexplored attitude toward the law, using an interview-based constructionist method on twelve teachers of Lvliang city in a Chinese K-12 context. The findings suggest that the participants prefer to acquire discipline knowledge by interacting with multiple power relations in their local environment and that their knowledge is historically and culturally specific. Although the disciplinary right they construct is never static, balanced, or essentialised, the participants’ constructions are commonly not in line with legal provisions or the aims of the law. Furthermore, school discipline legislation plays a silent role in empowering teachers to discipline students, but it is more visible in holding back teachers’ use of corporal punishment. These findings illustrate the complexity of implementing school disciplinary law as a universal national policy.

## Introduction

The challenge of instilling appropriate school discipline may differ across countries due to different legal and social contexts (Arum and Ford, 2012). In China, the *status quo* of K-12 school discipline is ambivalent. Some K-12 teachers use corporal punishment (CP) abusively, while others are reluctant to discipline students (Liu and Cheng, 2020; Liu and Zhang, 2020; Qin, 2020; Duan and Yang, 2021). To solve this problem, the Ministry of Education of the People’s Republic of China [MOE] (2020) issued a draft for public suggestions on implementing disciplinary rules for K-12 school teachers on 22 November 2019. On 1 March 2021, the disciplinary rules for K-12 education (trial implementation) came into force. Since then, disciplining students by Chinese teachers has been a legal right.

Different from many Western countries, the major aim of the legislation about how to discipline students is for creating a harmonious and orderly educating environment, revisiting traditional norm of respecting teachers’ authority (Zuo and Hu, 2012) and fixing the relationship between students and teachers (Xia et al., 2020). In modern Chinese context, “discipline” is not CP and is defined as “an educational behavior managed by schools and teachers to urge students who violate rules to correct their mistakes” (Ministry of Justice of the People’s Republic of China, 2019). The law classifies discipline into mild, moderate, and severe levels, according to the violation committed by the student and the circumstances thereof. Article 8 stipulates that every teacher has the right to use the following actions:

(1) Verbal reprimands;
(2) Order students to make an apology or an oral or written review;
(3) Appropriately add additional teaching or class public welfare service tasks;
(4) Order students to stand in the classroom during one class teaching time;
(5) Give after-class instruction;
(6) Use other appropriate measures stipulated by school rules or class conventions.

Furthermore, interpreted by Chinese society as the red line that teachers cannot cross, Article 12 lists eight prohibited measures:

(1) Using CP (i.e., hitting or stabbing) that causes physical pain;
(2) Punishing in disguised forms, such as standing above the normal limit, repeated copying, forced uncomfortable movements or postures, and deliberate isolation, which indirectly harms students’ physical and mental health;
(3) Abusing or violating students’ dignity with discriminatory and insulting words and deeds;
(4) Punishing all students because a few students violate rules;
(5) Punishing students for their academic achievements;
(6) Selectively punishing students due to personal emotions or preferences;
(7) Appointing some students to discipline other students;
(8) Other violations of students’ rights.

### Research of teachers’ practice of discipline

Although school discipline does not involve CP in some cultural contexts (Mamatey, 2010; Arum and Ford, 2012; Govender and Sookrajh, 2014), literature reviews on discipline cannot be divorced from the long-lasting phenomenon of CP in human society. Defined as inflicting physical pain on offenders to modify their misbehaviors (Wilson, 2002), CP is one of the oldest and most prevalent educational means adopted by almost every nationality, race, religion, and social class (Middleton, 2008; Brown, 2009; Durrant, 2020). In Anglo-Saxon literature, the doctrine of ‘*in loco* parentis’ historically gave teachers the considerable authority to punish children (Nelson, 1965; Pagliarino, 1970; Wilson, 1982; Segalo and Rambuda, 2018), and children were treated as ‘recipients of unquestioned violence in the child-adult relationship’ (Richardson and Wilcox, 1994, 175). In the eighteenth century, some French and English thinkers were against ‘the scholastic punishment’ (Durrant, 2020), and in 1839, the first parenting book to oppose CP was published (Chavasse, 2018). Between the 1820s and the 1850s, voices condemning physical punishment rose in the United States (Glenn, 1981). In 1867, New Jersey became the first United States state to abolish school CP (Thomas, 2020). At the beginning of the twentieth century, Western views shifted from seeing children as “property” (Hart, 1991; Richardson and Wilcox, 1994) to regard them as “essential human resources whose mature form would determine the future of society” (Hart, 1982, 4). In the 1940s, CP diminished and was abolished in the United Kingdom (Middleton, 2008). Since 1989, the UN Convention on the Rights of the Child has been advancing children’s rights worldwide (United Nations General Assembly [UNGA], 1989) and strongly advocating the self-determination rights of children (Hart, 1991). In 2006, the United Nations Committee on the Rights of the Child [UNCRC] (2006, 6) claimed, ‘all forms of physical or mental violence does not leave room for any level of legalized violence against children’. By 2020, 59 countries had fully prohibited CP in all settings, and 132 countries had banned school CP (Durrant, 2020).

However, the debate over CP is contentious. Many studies have demonstrated that CP is cruel, abusive, and humiliating, which encourages the replication of physical or psychological violence, discourages learning, and reduces learning passion and motivation (e.g., Ariès, 1962; Glenn, 1981; Hyman, 1990; Wilcox and Richardson, 1993; Ramsburg, 1997; Gershoff, 2002; Straus, 2003; Cicognani, 2004; Cameron, 2006), while other researchers contend that CP is effective in class management. Baumrind (1996) questioned the negative relationship between CP and psychological problems, and Wilson (2002) concurred that CP is fair for everyone because everyone dislikes pain. Naong (2007) reported that banning CP in South Africa weakened local schools’ discipline and frustrated teaching morale. Moreover, Arum and Ford (2012) argued that CP could result in respect for teachers, and students in countries with stronger teaching authority show better performance in math and science.

Interestingly, since the 21st century, countries such as the UK, South Korea, Singapore, Germany, Australia, and South Africa have legalized teachers’ right to discipline delinquent students (Wu, 2012; Department for Education, Government of the United Kingdom, 2016; Deakin et al., 2018; Segalo and Rambuda, 2018; Zhang and Wang, 2021). Globally, school discipline shows a common trend of being institutionalized (Deakin et al., 2018).

### The development of discipline in China

As a local and international trend, CP in China was also historically rife, condemned in the past century, and legally banned in recent decades. Based on hierarchical relationships, Confucianism entitled ancient teachers to the unquestionable power to punish students, which can be seen in ancient proverbs such as “beating is a sign of affection,” “a good beating makes the number one scholar,” and “a lenient teacher is an inert teacher.” These traditional beliefs were not challenged until the 1840s. After the Opium War, traditional Chinese agricultural society transformed into a semi-colonial and semi-feudal social structure. During this historical period of upheaval, away from Confucian tradition, a new intellectual class that embraced the Western worldview of science and democracy began to emerge, promoting the New Culture Movement (Wang, 2019) in the 1910s and 1920s, which questioned the authority of traditional Chinese teachers (Shi, 2019).

Furthermore, the Cultural Revolution between 1966 and 1976 refuted the traditional Chinese virtue of respecting teachers and damaged Chinese teachers’ social status (Zhao, 2014). After the Reform and Opening Up Policy in 1978, Western concepts were introduced into China again, such as children’s rights, the learner-centered teaching approach, and appreciative education (Yan, 1999; Huang, 2016; Li and Huang, 2020; Qian and Ma, 2020; Xia et al., 2020), thus greatly improving students’ rights and weakening strict education. In 1986, the Compulsory Education Law of the People’s Republic of China abolished CP in school systems. However, this law did not play a significant role in curbing CP, as many studies have demonstrated that CP is still widely practised by many Chinese teachers (e.g., Liu and Su, 1997; He, 2001; Wei, 2011; Yu and Gao, 2018). At the same time, school punishment creates conflicts between the family and schools (Zuo and Hu, 2012; Liu and Cheng, 2020; Shen, 2020; Wu, 2020). Current studies about school discipline conducted by Chinese researchers do not mostly focus on the development of children but on the return of teachers’ authority and the harmony of school management. It should be mentioned here that China’s One-Child Policy lasted 24 years (from 1982 to 2016) and made every single child the only hope of a whole family leading to the current child-centered Chinese education culture (Zhang and Wang, 2021). Gradually, parental involvement has taken on a powerful position to intervene in teachers’ occupational duties (Wu, 2020). To create a more orderly school environment, Chinese society is revisiting the Confucian norms of respecting teachers’ authority (Xia et al., 2020).

### Research of teachers’ knowledge of legislation

If teachers’ concerns are not addressed, the enforcement of changed discipline policies ‘is bound to fail’ (Naong, 2007). Therefore, it is important to understand teachers’ perceptions regarding school discipline legislation. Some related studies exist, although they are few. Some researchers have demonstrated that teachers’ beliefs about discipline differ from the legislation. For example, in Govender and Sookrajh’s (2014, 14) study, even though “teachers regulate disciplinary techniques according to legislation, some teachers still see value in using CP.” Lwo and Yuan (2011, 158) also found that participants in Taiwan are fully aware of laws but do not believe that alternative disciplinary measures can “increase students’ self-control and management.” This is also evident in Segalo and Rambuda’s (2018) study, which found that some South African teachers feel that alternative discipline strategies are usually ineffective.

Similarly, Brown (2009) reported that surveyed South Korean teachers perceived the law banning CP as a hindrance to teaching. Mamatey (2010) continued to explore what drives South Korean teachers to endorse CP as an educational system factor but not the teachers’ beliefs. Furthermore, some empirical studies indicated that teachers feel reluctant or fearful to exercise their right to discipline learners “because they might in doing so infringe on students’ human rights” (Rossouw, 2003, 2), or because the learners might counter-attack them and threaten the teachers’ safety (Cicognani, 2004; Segalo and Rambuda, 2018). Zuo and Hu (2012) assert that Chinese teachers are unclear about the word “appropriate” in the legal provisions; therefore, they have a wait-and-see tendency in practice. Skiba (2016) found that disciplinary right has been unequally exercised by United States teachers as marginalized students are more likely to be disciplined.

The above studies reveal a gap between practice and law, which indicates disparities between teachers’ grassroots knowledge and official knowledge. Previous studies have put forward the following suggestions: Instead of using coercive power, teachers should display expertise in their teaching to command authority (Tlhapi, 2015); class sizes should be reduced (Lwo and Yuan, 2011; Cheruvalath and Tripathi, 2015; Segalo and Rambuda, 2018); and teachers should “refresh their understanding of how discipline can be enforced and should work on findings new ways of effectively minimizing incidents of misbehavior” (Segalo and Rambuda, 2018, 5). Additionally, parental involvement should be reinforced (Segalo and Rambuda, 2018; Obadire and Sinthumule, 2021), and education administrators should supervise teachers to ensure they do not give up this right and include teachers’ exercise of the disciplinary right into the teachers’ performance assessment indicators (Yu et al., 2020). Educators should also make joint efforts to discipline mischievous learners (Obadire and Sinthumule, 2021). If teachers do not exercise their right to discipline students, this should be regarded as dereliction of duty (Yu and Gao, 2018; Li and Huang, 2020).

Moreover, teacher training about discipline should be strengthened (Lwo and Yuan, 2011; Segalo and Rambuda, 2018), and a standard procedure for resolving conflicts between school and family should be established (Lwo and Yuan, 2011). An institutionalized and transparent relief mechanism should also be built to safeguard students and teachers’ rights (Yu and Gao, 2018), and legislators need to standardize the formulation of school discipline plans and guide parents to participate in school supervision rationally (Zhang and Wang, 2021).

Nevertheless, many of the previous studies have adopted a “God’s eye view,” which regards teachers as a rational community. Second, some studies presume that the rights regulated by law equal the teachers’ real rights in practice. Third, suggestions for teachers are essentialist-oriented, paying much attention to the essence of teachers’ cognitive outcomes but overlooking how individual teachers’ constructions emerge and change during daily social interactions in specific contexts. As such, these studies have failed to provide a comprehensive and dynamic picture of teachers’ perceptions regarding their right to discipline students in the real world.

The phenomenon of school discipline in the modern world has been widely discussed in the existing literature. However, a discussion on the role legislation plays in school discipline is missing, and this role is fundamentally determined by teachers’ constructions, given that the test of authority should be its implementation at the micro-level (Foucault, 1980). Therefore, teachers’ constructing processes should be explored before offering them suggestions. This study is a step toward filling this gap in the literature.

### Theoretical framework: Social constructionism

Social constructionism is defined by Gergen (1973), who argued that knowledge is historically and culturally related and people must extend their enquiries into social, political and economic fields for a comprehensive understanding of the world. In addition to social conditions, Willig (1999a) insisted on the equal importance of individual action, which starts with people’s convictions about the nature of the society (Collier, 1998). Stressing cultural and historical specificity, social constructionism is also featured by anti-essentialism, which upholds the world as a product of social processes; thus, people have no essence or definable nature (Burr, 2003).

Additionally, social constructionists focus on “the dynamics of social interaction” (Burr, 2003, 9) and social practices that generate new knowledge (Foucault, 2000). As social interactions construct “our versions of reality” (Burr, 2003), people’s constructions are non-objective and partial (Jovchelovitch, 2001). Furthermore, constructionism studies emphasize the process of social construction, which is explained by Gergen (2014, 1772) as “negotiated agreements among people.” This emerging process is closely connected with iniquitous power relations, which lead people to make decisions (Burr, 2003).

Social constructionism also suggests that constructions are driven by language on the significance of “the social meaning of accounts and discourses” that reveal the “power relations operating in society” (Burr, 2003), and discourse analysis often attracts social constructionists’ interest. Knights and Morgan (1991) claimed that discourse is not just about how we describe the world but also how it influences people’s actions. Consequently, the constructive analysis often takes the form of the traditional qualitative interview approach, but the difference ‘is in the theoretical assumptions that are driving the analysis’ (Burr, 2003, 174).

Considering school discipline, people’s constructions regarding punitive violence have fluctuated with changes in economic, cultural, and social structures. The construction of individual teachers is worth exploring further. Kelly (1955) argued that every individual has unique ways of constructing the world, and understanding different constructions may lead people to create new possibilities for action. Subsequently, this study addresses the following questions:

(1) How do teachers construct their legislative right to discipline students?
(2) What are their constructions about this right?
(3) What role does the legislation play in K-12 school discipline?

## Methodology

### The sample and data collection procedure

Lvliang is an underdeveloped mountain city, located in the North central region of China. Adopting the snowball sampling method, the researchers designed semi-structured interview questions (See Table 1) revolving around teachers’ construction about the legal right and recruited 12 interviewees. Table 2 summarizes the profiles of the 12 participants who were recruited for the interview.

**TABLE 1:** Sample of the semi-structured interview questions.

The first round
Q1: Have you ever paid attention to teachers’ right to discipline students? How do you understand it? Q2: What do you think is the greatest difficulty of exercising this right in practice? Q3: Do you work in a school where there are many disciplinary phenomena for students as a whole? What kind of punishment do teachers usually use? …
**The second round**
Q1: What is the attitude of the teachers in your school toward learning the disciplinary rules for K-12 education? Does the principal attach importance to this? Q2: Do you think teachers in your school dare to discipline students since the implementation of the law? Q3: Has the principal’s management orientation changed? …

**TABLE 2:** Profiles of the participant group.

Participants	Gender	School type	Age	Teaching experience
A	Female	Public High School	59	34 years
B	Female	Private Elementary School	56	37 years
C	Female	Public Elementary School	46	27 years
D	Female	Public Elementary School	36	14 years
E	Female	Public Junior High School	32	12 years
F	Female	Public High School	45	24 years
G	Female	Public Junior High School	39	16 years
H	Female	Public Elementary School	34	12 years
I	Female	Public Elementary School	43	22 years
J	Male	Public Elementary School	38	15 years
K	Female	Public Elementary School	30	5 years
L	Female	Private Elementary School	31	9 years

The data collection lasted for more than one year. Two rounds of in-depth interviews (i.e., 24 interviews) were conducted in Mandarin Chinese and were audio-recorded, transcribed, and then coded by Nvivo 12. The first interview round took place in July 2020, nine months after the draft of public suggestions was issued by the Chinese Ministry of Education, mainly focusing on the teachers’ construction process about the right to discipline students. In July 2021, four months after the formal implementation of the legislation, the second interview round was conducted, which mainly concentrated on teachers’ perceptions of the new legislation. Additional questions were asked within the predetermined thematic framework based on the participant answers in order to allow the researchers to develop a keen understanding of participants’ thoughts. As social constructionists call for a democratic research relationship (Burr, 2003), researchers employ the approaches of “collaborative inquiry” (Gergen, 1999) and “community psychology” (Orford, 1992) to seek “participant-led ways of improving specific social situations” (Willig, 1999b, 7) during interviews. Moreover, because social constructionist research never aims to identify a final description of the world, reliability and validity are not suitable for evaluating the quality of social constructionist research (Burr, 2003).

### Data analysis and presentation

As the social constructionist perspective rejects ‘objectivist’ vision of social groups but emphasizes the constructing process of realities through emerging social interaction (Dervin, 2011), the initial data analysis resulted in a large cluster of data linked to various interacting ways. Notably, every individual teacher’s constructing process and results are never formed by solely interacting with just one party from the groups of parents, principals, colleagues, students and the social environment. Instead, the interaction process often takes place in a non-liner way. Therefore, the coding process involved an interaction between examining the data of multiple interacting ways and referring to theories linked to social constructionism. Guided by the social constructionism theory, the second round of data analysis looked for data relevant to the “priori codes” (Johnson and Christensen, 2018, 489) of “negotiating identity,” “private conversation,” “observation,” and “personal experience” that were identified before doing the analysis. Also, the inductive code of “interculturality” was generated during the data analysis process (See Table 3).

**TABLE 3:** Sample of the coding process.

Interview data	Codes	Priori code	Inductive code
Now, teachers often say that parents are God!	Negotiating identity	√	
She kept telling me never to touch any delinquent students	Private conversation	√	
Our principal merely read this law to us but never truly encourages us to discipline students as he does not want to cause problems for himself.	Observation	√	
…“what teachers can do” regulated by the new legislation does not work well in real class management.	Personal experience	√	
I hear that CP is legal in America. Why are we just learning appreciation education from them?	Interculturality		√

Even though the research questions tend to address K-12 teachers’ “constructing ways,” “constructing results,” and “constructing influences,” the latter two themes are always co-occurring with the first one. Therefore, the finding part will in details explain the concrete interacting process that how participants constructed their knowledge about the right to discipline students, and the discussion part will address all of the three research questions.

## Findings

### Finding 1: Constructing from negotiating identities

Primary socialization “involves being an identity and a place in society” (Andrews, 2012, 41). Identity “originates not from inside the person, but from the social realm, a realm where people swim in a sea of language and other signs’ (Burr, 2003, 109). How do people describe the reality indicates hidden power relations (Burr, 2003). Teachers in this study commonly construct and negotiate their “fragmented, shifting and temporary identity” (Burr, 2003, 54) as disadvantaged roles during family-school interactions, of which the process is often filled with conflicts (Burr, 2003). Additionally, with the prevailing use of mobile Internet technology, online chat groups have become a normalized communication channel between school and family. By taking advantage of communication technology, the online “self-organization” (Paul, 1988) of parents comes into being, opening up previously closed classrooms to the public. Participant C expressed the teachers’ concerns as thus:

Now, teachers often say that parents are God! Every class has a WeChat group (a popular mobile phone chat tool in China), and parents also set up WeChat groups that exclude teachers. This allows parents to interact with each other regarding teaching in school. The multi-media and We-media might focus their cameras on you… We dare not exercise this right entitled by the Ministry of Education. Who knows the result after punishment?

C seemed to suggest that parents stand in a higher position within school-family power relations. The metaphor of *God* implies the infiltration of consumerism culture into Chinese education, which enables one consumer (parent) to refer to another (Baudrillard, 1996). Also, she believes the technology tools weaken teachers’ authority and her attitudes toward the legislation are negative. This demonstrates people’s action is determined by the prevailing knowledge in the society (Burr, 2003) but not the legal knowledge. “Who knows the result after punishment?” reflects individual teachers’ fearful emotion as the result after exercising the disciplinary right is still unpredictable. Even though the legislation protects teachers’ right (if the teacher was the injured party), teachers think the appealing procedures are troublesome in taking time and energy. They don’t believe this legislation is capable of recalling their courage to discipline students. G negotiated teachers’ identity as a “vulnerable group.” She had this to say:

We teachers are very vulnerable when dealing with the administrative power. If this kind of things happen (family-school conflict), teachers are always those who are blamed for. Can I appeal to the law? But I still need time to work and earn money.

Although the legislation frames the disciplinary right, teachers tend to seek for other ways of strengthening their rights. C has also constructed that trust from parents can reinforce teachers’ disciplinary rights through interacting with many parents. To her, improving children’s test scores has become a means of self-empowerment. She felt confident about constructing an identity of the “model teacher’ to win parents” trust and more real rights to discipline students:

I am a model teacher and always play a leading role among colleagues. How do you make them (parents) trust you? The first thing is to improve their children’s testing scores. Those teachers who are bad at that are very likely to trigger school-family conflicts.

### Finding 2: Constructing from private conversations

Burr (2003) asserts that our understanding of the worlds is never objective, which comes from people around us. Berger and Luckmann (1991) maintain that conversation maintains, modifies and reconstructs subjective reality. It was found that no participants in this study had carefully read the legal documents about discipline, but they would like to obtain the objective knowledge from private conversations among colleagues. The shared inner thoughts and subjective experience serve a more constructive role in building their knowledge. For instance, from participant G’s viewpoint, the role of legislation is invisible in empowering teachers with disciplinary rights but visible in curbing teachers’ punishment behavior and lowering teaching morale. Her construction arose from a private dialog, from which she internalized the legal effects by understanding how the authorities representing the law deal with ordinary teachers. Then, a negative attitude toward the law has been passed on to others.

Recently, an experienced teacher in our school was forced to suspend a class. Out of responsibility, she used CP on students, and the parents complained against her. Eventually, even the superintendent came to our school to deal with her case. I feel bad and angry. She told me that those who do nothing about discipline have no problems with their teaching careers, but being responsible will stir up trouble. I felt her depression. She kept telling me never to touch any delinquent students.

Also, all of 12 participants shared the family-school conflicts aroused by punishment, which just happened around them. Actually, teachers talk to each other about those incidents in their daily life, invisibly building up teachers’ knowledge about the law from a bottom-to-up pattern. Those individual teachers are not only information receivers but also information deliverers. From this research, the legislation doesn’t change teachers’ original conviction. They are inclined to draw informal working experience about how to avoid troubles in teaching practice through private conversations. For instance:

I often told young teachers that, if you don’t discipline students, the worst thing is that you are seen as a bad teacher. But it is better than losing your career. Right? If conflicts happen, you are going to be blamed by the whole society (A).

I am sure no parents in nowadays encourage you to beat their children. Even though a few parents told teachers to discipline their kids, we would not do that. Many colleagues told me those parents didn’t say what they really thought. If you really discipline their kids, you might be complained (J).

### Finding 3: Constructing from observing the education environment

Social constructionists believe that people’s observations reflect the real world (Andrews, 2012). Teachers can directly observe if the law has changed the education environment. All 12 participants reported that their school principals organized conferences to help teachers understand the new legislation about organizing their right to discipline students. Moreover, they commonly confessed that they paid little heed and trust to the principal’s public discussions about the new legislation. However, according to the interview data, not all participants could clearly distinguish between CP and discipline. Many of them observed that their colleagues did not care about the new law. Their commonly indifferent learning attitude resulted in the formalism of the legislation. Additionally, China is a high-context country; thus, teachers tend to sense the regulatory climate from the principals’ actions rather than the principals’ public discourses. For example, Participant H said,

Our principal read this new law to us. But I don’t think she really attaches importance to it. When it comes to discipline, we still associate it with beatings. My colleagues and I never actively acquire legal knowledge on our own unless we hear complaints from parents. Nothing has changed. CP is still commonly seen in my school.

It can be seen from this micro environment that the intention of principals’ public talk was supposed to impart the knowledge of legal rights. Nevertheless, teachers observed that principals also played a passive role when facing complaints from parents. This observation in fact decreased teachers’ willingness to study the law. Participant B tried to explain why teachers were not concerned about the new legislation. She posited that, from the principal’s perspective, encouraging teachers to discipline students might threaten the safety of the school management. B commented on the principal’s blocking role as follows:

Our principal merely read this law to us but never truly encourages us to discipline students as he does not want to cause problems for himself.

### Finding 4: Constructing from subjective disciplinary experience

The world can never be known if human subjective experience is neglected (Andrews, 2012). In this study, participants are likely to justify the feasibility of disciplinary measures offered by legislation by reflecting on their subjective practices. Whether rational or emotional, teachers’ reflection is certainly a mode of thinking (Dewey, 1933), often stimulated by teaching practice, which is supposed to “be a powerful tool contributing significantly in maximizing teacher learning and their professional expertise” (Stavroulia and Lanitis, 2020, 286). Some identified scenarios evolve around teachers’ practice of discipline is teachers’ reference to legal policies when they need to make “disciplinary decisions” (Yell and Rozalski, 2008).

Disciplinary practice in schools is more complex than the formal written legislation that governs it on the surface. The main role of the legislation is to guide the school to have the intended view of what it wants it to be. Its effectiveness is to be perceived as fair for all students, protecting students’ right to education in a safe and dignified environment. However, in schools, the relationship between the legislation and practices tends to be of a nominal value. There is no doubt that teachers undertake efforts to comply with legislation, they reflect the use of more creative ways to create school norms that are more relevant in addressing behavioral issues. Nevertheless, this study finds that teachers’ subjective experience only helps them construct the idea that the legislation does not facilitate a good learning climate. This is consistent with the idea of Hammersley (1992) that reality means the subjective experience of every day rather than the objective world. For example, Participant J said the following:

We are not allowed to use CP now, but “what teachers can do” regulated by the new legislation does not work well in real class management. For example, ‘standing against the wall as a punishment’ hardly subdues students, especially younger pupils.

### Finding 5: Constructing from interculturality

China is ‘an extremely diverse country’ (Dervin, 2021, 35) with an enormous population and a vast territory, of which the social homogeneity brought about by industrialization and commercialization is lower than that of small developed countries (Su, 2011). Even though the same legislation presides in all places for K-12 teachers in China who share the same passport, teaching conventions, and official language, implementing the same policy is heterogeneous because of cultural distances. Many participants in this study think that Lvliang is a small mountainous city, often reacting slower to national policies than developed areas. Based on an intracultural perspective, participant B had this to say:

I think (teachers) in big cities might be better (in knowing the documents). In small cities, teachers are just either busy with teaching or household duties. They don’t pay attention to the new law.

Participant A’s account demonstrates an essentialist tendency of trying to catch the “cultural essence” (Dervin, 2011) of cultural groups in different nations, as she integrated critical cultural awareness into her construction of the law. Her “skills to critically evaluate practice and products of one’s own culture and those of other cultures” (Byram, 1997, 53) led her to make a cultural comparison, thinking about the differences and similarities, and formed her critical attitudes toward the law:

You see, when Westerners make hamburgers, they devote particular attention to standardized production, but traditional Chinese chefs never use a measuring glass or weighing machine. The food-making process was not normalized, but Chinese food tastes great!

CP had been an undoubtful teaching act until Western concept of children’s rights was introduced to China. Since then, voices for respecting children’s rights have increased, which influence people’s understanding and practice of disciplining. Participant B criticized that the over-emphasis on appreciation education was not good for children’s development. She believed the return of strict education was very necessary. When she shared her cognition about the right to discipline students, she recognized that education culture was fluid and showed some cultural ethnocentrism.

You see, we learn the appreciation education from USA. I hear that CP is legal in America. Why are we just learning appreciation education from them? They just don’t let us learn the right thing (strict education)!

## Discussion

### How do teachers construct their legislative right to discipline students?

Regarding the first question, the findings demonstrate that while interpreting the law, teachers are inclined to acquire their subjective knowledge from interacting with the small world they inhabit. For them, official ways are not the only approach to understanding the extent to which they can exercise their right to discipline students in practice. Instead, after interacting with students, parents, colleagues, and principals in different micro contexts, teachers are like rhetoricians, using their capacity for identity negotiation, argument, justification, and criticism but not absorbing the literal rules without reflection (Billig, 1987). These findings echo Sarbin’s (1986) opinion that human beings often impose a structure on personal experience, and this structure present both their experience and how they represent those to themselves.

Daily interactions constitute individual teachers’ working experience, construct teachers’ underlying beliefs about their positions in a set of non-linear power relations, and “reaffirm the basic validity of this dominant moral order” (Kitzinger, 1989, 95). The findings regarding the participants’ constructing process demonstrate that what motivates teachers to shift their attitude is not top-down training but the sense of safety from micro contexts. This finding raises questions about the suggestions made by Segalo and Rambuda, who stressed teachers’ agency and ignored that individual teachers’ cognitive process is often in the service of their own interests (Burr, 2003). Furthermore, in Participants B and H’s construction processes, principals do not truly value the discipline legislation. This finding reveals school administrators’ inner attitudes toward the law, which contrasts Yu et al’s. (2020) suggestion that education administrators should supervise and evaluate teachers to ensure they do not give up their disciplinary rights.

Moreover, education is a moral and ethical act (Reagan, 1993). As emotional human beings with moral senses who “make large personal emotional investments in their practice’ (Juan, 2018, 1), the participants” constructing processes are accompanied by emotions and moral sense. From this study, in disciplinary settings, teachers construe that anger and regret toward disobedient students occur out of a sense of responsibility and love. Except for taking personal interests into account, the findings demonstrate that teachers need to be praised, affirmed, and recognized for their moral behaviors as their disciplinary acts are not intended to cause harm. Discipline is, therefore, a kind of emotional and moral labor in some participants’ constructions. When students and parents misunderstand this labor, they feel frustrated, helpless, and even scared of their duties. Therefore, the suggestion that taking no disciplinary measures means a dereliction of duty (Yu and Gao, 2018; Li and Huang, 2020) might aggravate teachers’ negative emotions, which “can only fast track their exit from the profession” (Naong, 2007, 297).

### What are their constructions about the disciplinary right?

In terms of the second question, the data demonstrate the uniqueness in participants’ constructions of the law on discipline. Their constructions are subjective, fluctuating, and unbalanced. The constructions include the following: discipline means CP; beating is a sign of being responsible; the disciplinary right in law is ineffective in practice; to improve students’ test scores, teachers must be empowered to discipline the students; the teachers’ right to discipline is limited and not encouraged by the law; and giving up the disciplinary right is for their occupational safety. Even though the findings of K-12 teachers’ construction results are individualized, in some way, they reflect Chinese history, cultural norms, educational values, and economic structure.

In China, CP has traditionally been viewed as necessary, and its recorded history can be traced at least 2,500 years back (Tang, 2017). It also includes a long history of adopting Confucian doctrine to dominate the classroom moral order, but the traditional culture that teachers should be honored, respected, and not doubted has eroded in the globalization era. With frequent interactions with Western cultures, modern China is moving away from its traditions, becoming increasingly similar to developed countries in the West (Su, 2011). Mayo (1945) points out that traditional society is stereotyped, as the modern industrial world is a society that requires adaptation to rapid changes. Consequently, conflicts between the old and new cultures in Chinese society have intensified. Over the past 40 years of the Reform and Opening Up Policy, the eastern regions of China have taken the lead in industrialization, but the central and western regions have developed slowly. Accordingly, the degree of industrialization and legalization in undeveloped areas, such as the mountainous city of Lvliang, is lower than that in many developed cities in the easter part of China. The traditionally qualified privilege to inflict CP on learners is easier to replicate in these backward areas. This finding might explain why teachers in low-economy areas still associate discipline with CP, which also supports Durrant’s, 2020 conclusion (2020, 9) that CP is more prevalent “in countries with higher levels of inequality and lower levels of democratic decision-making.”

Confucianism links academic achievement to personal success (Hesketh and Ding, 2005). Traditionally, Chinese people believe that ‘to be a scholar is to be the top of society.’ With the rapid economic development of mainland China, competition for jobs has become increasingly fierce (Hesketh and Ding, 2005; Dello-Iacovo, 2009). Unfortunately, Chinese parents’ assessment of teachers tends to be homogenous: Students’ academic achievement speaks louder than anything else. This alienated evaluation is intensified by consumerism (Lin and Lu, 2020), and modern technological communication offsets the boundary between school and family. Tlhapi (2015) proposed that teachers win their authority by showing their expertise. However, the findings of this study demonstrate that some teachers tend to construct this “expertise” as the teacher’s capability to raise students’ grades. This construction cannot be dissociated from the contemporary Chinese educational context, emphasizing academic achievement.

Many Chinese scholars report the legal mechanisms of countries such as the United Kingdom, the United States, Japan, or Germany for reference. However, from the findings of this study, transplanting the legalism experience is not a viable way to help teachers feel less ill-equipped to handle discipline problems in underdeveloped areas like Lvliang, where the legislative disciplinary right lacks a solid legal foundation and social support. In this globalized, industrialized, and informational era, disciplining disruptive students has become a legal concept, while punishing students had been a non-institutionalized personal behavior embedded in China’s long history (Zhang and Wang, 2021). The conventional identity of teachers was not that of a law executor, and their disciplinary behaviors were never standardized before this law came out. Participants’ constructions also reveal that it is still hard for teachers in Lvliang to understand the ‘separation of law and morals’ (Hart, 1958, 598).

### What role does legislation play in school discipline?

The reality is socially defined by groups of individuals (Berger and Luckmann, 1991). Teachers’ indifferent attitudes toward the official knowledge are detrimental to the landing of the disciplinary legislation. Albeit based on varying constructions shaped by different experiences, the participants in this study have little interest in familiarizing themselves with newly promulgated legislation. When they have convincing reasons to punish students, they are willing to enforce discipline constructed through dynamic interactions rather than legal instruments. Teachers only recognize the compulsory power of the law when severe family-school conflicts take place. Therefore, legislation plays a silent role in K-12 school discipline in Lvliang.

First, this legislation aimed to ensure and standardize teachers’ implementation of their legal duty in managing students and maintaining their own dignity (Ministry of Justice of the People’s Republic of China, 2019). In this sense, the new legislation is assumed to regulate and rebuild the relationships between schools and families. Notably, this law merely outlines the red line for teachers, but parents’ behavior is still unrestricted. This means that the legislation, indeed, increases the definiteness of disciplining means, but what will happen after disciplining is still unpredictable. Avoiding discipline, therefore, seems to be self-protective for personal interests, which eventually leads to the “tragedy of the commons” (Hardin, 1968, 1243). This echoes the opinion that possibilities for action come with ‘identity and power implications’ (Walkerdine, 1981) rather than “the essentialist connotations of personality” (Burr, 2003).

Second, principles put forward by law are general and essential, and cannot contain specific cases (Lerner, 1989). Due to diverse disciplinary settings and experiences, teachers’ constructions of their legal rights are pluralistic, fluid, and anti-essential. Furthermore, the essence of teachers is impossible to define because teaching staff comprise individual teachers with individualized experiences and professions. Even though a group of teachers stays in the same cultural environment, they have a non-essentialist interpretation of the official policy. Therefore, presupposing a unified execution of the official policy is formidable, especially in such a large country.

Last, the participants are not used to depending on rational and standard provisions to discipline students, which makes sense in the Chinese cultural context. Although one participant’s example of making food indicates an essentialist perspective of seeing Chinese as a homogeneous group, the silent role of legislation in K-12 schools in Lvliang, to some extent, has its roots in the Confucian idea that law cannot govern everything (Matthyssen, 2021). Adherence to law has traditionally been absent in ancient China (Weatherley, 2002; Hansen, 2004). This sort of reluctance toward legislation left much room for long-lasting arbitrary interpretation (Matthyssen, 2021). Even today, acting meticulously according to the law in Chinese society is often considered dogmatic (Su, 2011). As products of Chinese culture and history, the participants still think that discipline is a form of tacit but not legal knowledge.

## Conclusions and implications

Within the framework of social constructionism, social change is made by human activity (Berger and Luckmann, 1991). Teachers’ subjective constructions regarding the right to discipline students are based on multiple non-linear interactions with every stakeholder living in the same environment. The inculcation of morals and values cannot rest solely on the shoulders of teachers because the dilemma of school discipline in China is co-constructed by a network of complicated power relations as heretofore discussed, such as school and family education, legal history, traditional Confucianism, industrial transformation, modern rational orders, globalization, cultural change, national policies, and technology. The collective action predicament of teachers can never be tackled by elaborately manipulating any single side of them. Teachers in Lvliang lead a passive and surface learning attitude toward the new disciplinary legislation for cultural, historical, and social reasons. In conclusion, legislation plays a silent role, but it is more visible in suppressing teachers’ use of CP rather than in encouraging teachers to discipline students.

Although social constructionism is very limited in suggesting an alternative way of life after showing the identity and power relations (Burr, 2003), this study contributes to a holistic picture of the neglected disciplinary right that teachers construct in daily life and their underexplored attitude toward the law. The following are suggestions for future research:

(1). Teachers’ tacit knowledge of the appropriate discipline in teaching settings should be observed, collected, and transformed into teacher training by experts. Therefore, scholars and policymakers should enter the real world of K-12 teachers to seek alternative disciplinary measures in consultation with teachers.
(2). Principals significantly influence the legal and regulatory climate of school discipline, but this study found that they play a blocking role in exposing the legislation to teachers. Thus, there is a need for further research on the concerns and attitudes of principals toward the same issue.
(3). The legislation on school discipline regulates what school insiders can and cannot do. However, parental behavior or involvement is not regulated by law. Therefore, as stakeholders, parents’ construction of school discipline should be explored.

## Data availability statement

The datasets presented in this study can be found in online repositories. The names of the repository/repositories and accession number(s) can be found below: https://figshare.com/articles/dataset/Interview_Data_nvp/19243977.

## Author contributions

MB was mainly responsible for the literature review, theoretical framework, research design, data collection, and data analysis. GO helped the MB in discussing the findings and polishing the written language. MB and GO joint efforts in completing this manuscript. Both authors contributed to the article and approved the submitted version.

## Conflict of interest

The authors declare that the research was conducted in the absence of any commercial or financial relationships that could be construed as a potential conflict of interest.

## Publisher’s note

All claims expressed in this article are solely those of the authors and do not necessarily represent those of their affiliated organizations, or those of the publisher, the editors and the reviewers. Any product that may be evaluated in this article, or claim that may be made by its manufacturer, is not guaranteed or endorsed by the publisher.
